# Bio-Sourced Flame Retardants for Textiles: Where We Are and Where We Are Going

**DOI:** 10.3390/molecules29133067

**Published:** 2024-06-27

**Authors:** Giulio Malucelli

**Affiliations:** 1 Politecnico di Torino, Department of Applied Science and Technology, Viale Teresa Michel 5, 15121 Alessandria, Italy; giulio.malucelli@polito.it; Tel.: +39-0131229369; 2 Consorzio Interuniversitario Nazionale per la Scienza e Tecnologia dei Materiali (INSTM), Via G. Giusti 9, 50121 Florence, Italy

**Keywords:** textiles, flame retardancy, flame retardants, bio(macro)molecules, flammability, combustion behavior, intumescence, charring, durability

## Abstract

After the period of halogenated compounds, the period of nano-structured systems, and that of phosphorus (and nitrogen)-based additives (still in progress), following the increasingly demanding circular economy concept, about ten years ago the textile flame retardant world started experiencing the design and exploitation of bio-sourced products. Indeed, since the demonstration of the potential of such bio(macro)molecules as whey proteins, milk proteins (i.e., caseins), and nucleic acids as effective flame retardants, both natural and synthetic fibers and fabrics can take advantage of the availability of several low-environmental impact/“green” compounds, often recovered from wastes or by-products, which contain all the elements that typically compose standard flame-retardant recipes. The so-treated textiles often exhibit flame-retardant features that are similar to those provided by conventional fireproof treatments. Further, the possibility of using the same deposition techniques already available in the textile industry makes these products very appealing, considering that the application methods usually do not require hazardous or toxic chemicals. This review aims to present an overview of the development of bio-sourced flame retardants, focusing attention on the latest research outcomes, and finally discussing some current challenging issues related to their efficient application, paving the way toward further future implementations.

## 1. Introduction

Textile materials play a key role in our everyday lives, from domestic [1] to industrial uses [2]. However, since textiles are mainly composed of polymers, they can easily ignite and burn when exposed to an irradiative heat flux or a direct flame, if not intrinsically fireproof. Apart from the hazards and dangers derived from the heat evolved during combustion because of the exothermicity of the combustion reactions, the high flammability of these materials poses some safety issues as the smoke generated can be harmful and impede the escape from the area involved in the fire accident.

In 2023, the International Association of Fire and Rescue Services documented the generalized data on the situation with fires in the world from 1993 to 2021, elaborating the statistics concerning 38 countries all over the world and highlighting about 3.1 million fires, 16,800 fire deaths (1.3 every 100,000 inhabitants), and more than 62,000 fire injuries (4.9 every 100,000 inhabitants) [3]. In particular, the report evidenced that inhalation of smoke and toxic combustion volatiles (especially CO) represents the main cause of fire deaths, while exposure to the evolved heat mostly accounts for injuries. With particular attention to textile materials, the UK Government [4] reported the occurrence of about 760,000 fire incidents, most occurring in the home and encompassing nightwear, bedding, and upholstered furniture. 

To decrease and limit the issues related to fire, several flame retardants (FRs), differing as far as their structure, chemical composition, and flame retardant mechanism are considered, have started to be developed. FRs are capable of lowering the risk of fire, either by inhibiting the material’s ignition or by slowing down the flame propagation when needed [5,6,7,8,9,10].

Specifically regarding textiles, fireproof materials can be designed and manufactured by directly introducing FRs into synthetic fibers through melt compounding and subsequent spinning processes. In this context, such reactions as copolymerization or grafting to the polymer backbone can be successfully exploited, though the number of suitable fibers is limited to the synthetic ones. Conversely, very often, surface-engineered strategies, which are effective on both natural and synthetic fibers and fabrics, are employed. In this regard, the textile surface can either be coated with a discontinuous or continuous flame retardant layer or impregnated in a bath containing a solution or suspension of the FR additive, achieving the envisaged wet pickup [11,12,13]. 

A nice review paper from Horrocks [14] proposed a rational classification of the FRs for textiles according to three different periods, namely: the “golden” period (roughly from 1950 to 1980), which showed the appearance of the first patents on organophosphorus-based products, as well as the manufacturing of intrinsically flame retarded synthetic fibers bearing aromatic groups; the “1980s–late 1990s” period, during which the novelty of the performed research was quite limited; finally, the “2000 onward period” characterized by numerous investigations regarding the design of char-promoting and intumescent FRs, possibly in combination with P- or P/N-based additives for the development of synergistic effects.

It is precisely during this last period that academia started to think about the use of bio-sourced/bio-based products as potential flame retardants for polymeric materials and, in particular, for textiles. Indeed, several bio-based products have a chemical structure and contain the elements (such as P, N, S) usually employed for the design and synthesis of conventional flame retardants and, therefore, can be useful for effectively protecting fibers and fabrics from the application of a direct flame or exposure to an irradiative heat source [15,16,17].

**Figure 1:**
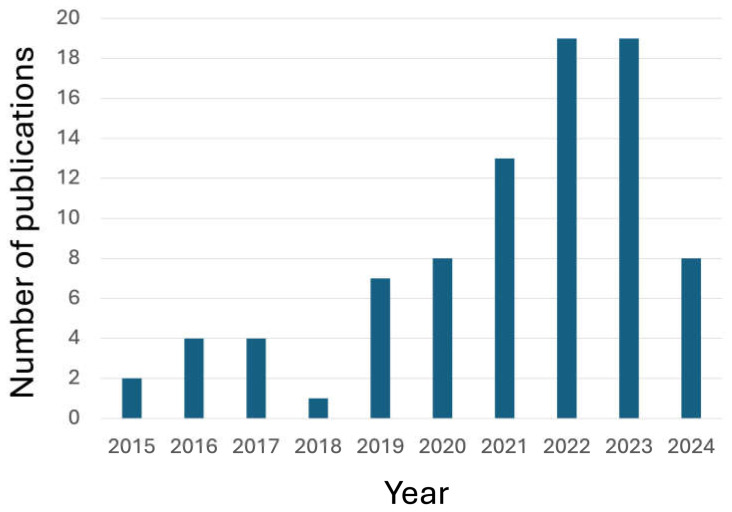
The number of publications (from 2015 to 2024) in peer-reviewed journals, dealing with “Bio-based AND Flame retardants AND textiles” (AND is the Boolean operator; data collected from the Web of Science^TM^ database, www.webofscience.com, accessed on 19 June 2024).

The scientific interest in the use of these bio-sourced flame retardants is well documented by the increasing trend in the papers published in peer-reviewed journals during the last ten years (Figure 1).

The present review aims to discuss the current state of the art related to the use of bio-sourced flame retardants, specifically for natural and synthetic fabrics. First, a general classification of the bio-sourced products with potential flame retardant features will be provided; then, an overview of the continuous progress (during the last four years) on the design and implementation of bio-based FRs for textile materials will be proposed. Finally, their current limitations will be discussed, highlighting some perspectives on possible research trends for the next few years. 

## 2. Classification of Bio-Sourced Flame Retardants or Bio-Sourced Biomasses as Building Blocks for the Design of Flame Retardants

**Figure 2:**
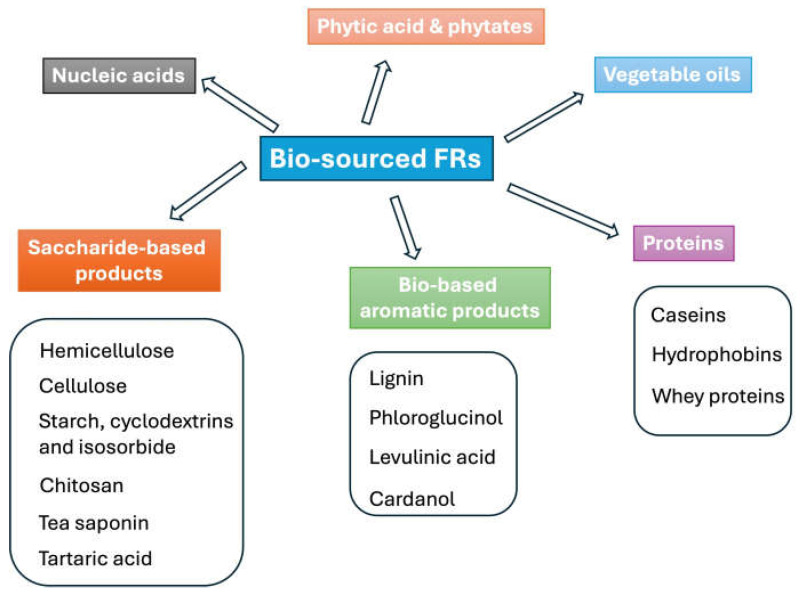
Classification of bio-sourced flame retardants.

A general and simple classification of bio-sourced flame retardants (not specifically devoted to textiles) can be based on the type of biomass, from which they can be derived/extracted (Figure 2). 

### 2.1. Saccharide-Based FRs

Cellulose, a fibrous constituent present in the cell walls of higher plants, algae, bacteria, and fungi, is the most copious biopolymer in nature, with around 10^11^ tons produced per year [18]. It is also responsible for fixing around half of atmospheric carbon dioxide through photosynthesis [19]. As a saccharide-based biopolymer, cellulose consists of glucose units linked to each other to form long macromolecular chains with a polymerization degree (up to about 40,000), which strictly depends on the treatment (chemical, mechanical, or biological, among others) selected for its recovery and on the type of pristine biomass source. Moreover, the possibility of organizing its macromolecular chains in a 3D-ordered structure makes cellulose a semicrystalline polymer. This structure is stabilized through the occurrence of weak bonds (namely, van der Waals and H-bonds), as well as by exploiting hydrophobic interactions taking place among the polymer chains. As a result, cellulose fibers are formed by the microfibrils that, in turn, originate from the 3D structure of the biopolymer.

The huge number of reactive hydroxyl groups in cellulose (in C-2, C-3, and C-6 positions) accounts for its ease of functionalization, usually carried out as an esterification reaction. For instance, both the thermal stability and fire behavior of cellulose can be remarkably enhanced through phosphorylation, using different types of co-reactants (such as phosphonic acid, phosphorous acid, phosphoric acid, or phosphoric acid salts, as well as polyphosphoric compounds, among others); further, the grafting of phosphorus-containing polymers onto cellulose is a successful strategy [20,21,22]. 

Low molecular weight and highly branched chains of various sugars, which can be recovered from plant cell walls of biomasses through treatments with an aqueous alkaline solution, compose hemicellulose. In particular, hemicellulose consists of different five-carbon (such as *L*-arabinose and *D*-xylose) and six-carbon (*D*-mannose, *D*-galactose, and *D*-glucose) sugars and uronic acid. The quite low decomposition temperature (at about 180 °C) somehow limits the use of hemicellulose as a component of flame retardant recipes [23].

Amylose, a linear polymer based on glucose units, and amylopectin, a highly branched polymer consisting of glucose units bonded to the main glucose chain, are the main components of starch, a semicrystalline polysaccharide [24,25]. There are several methods for recovering starch from proteins and other compounds, which are carried out after mechanical treatments directly performed on such plant biomasses as roots, tubers, or seeds. They involve the use of aqueous solutions of NH_4_OH, Na_2_CO_3_, NaOH, and SO_2_, among a few to mention [26]. As far as flame retardancy is considered, starch was mainly devoted to the design of intumescent systems, exploiting its suitability as an effective carbon source [27]. Briefly, an intumescent flame retardant system consists of a carbon source, an acid source (that promotes dehydration reactions, favoring the formation of a polyaromatic stable protective char, also at the expense of the carbon source), and a blowing agent (that, upon degradation, favors the formation of gaseous products, responsible for the swelling of the newly formed char) [28]. Moreover, the ease of being oxidized toward the formation of salts of polyoxyacids widens the possibility of using starch as an effective flame retardant; in fact, oxidized starch decomposes between about 150 and 280 °C, giving rise to the formation of a foamed char that exhibits remarkable thermal shielding features, hence protecting the underlying polymer [29].

Isosorbide is a diol derived from sucrose. Apart from the interest gained as a potential replacement for bisphenol A in the synthesis of polycarbonates [30], isosorbide is easily converted into flame-retardant polyphosphonates (through direct polycondensation with phenylphosphonic dichloride) or phosphorus-containing esters (through reaction with castor oil derivatives).

**Figure 3:**
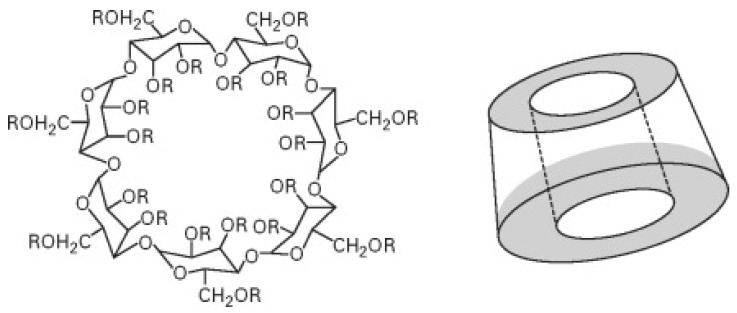
Chemical structure of β-cyclodextrin. Reprinted with permission from [33]. Copyright 2007, Elsevier.

Cyclodextrins represent a family of cyclic oligosaccharides made of six α-cyclodextrin, seven β-cyclodextrin, eight γ-cyclodextrin or more glucopyranose units linked by α-(1,4) bonds [31]. As previously observed for starch, cyclodextrins can also be exploited as an effective carbon source in flame-retardant intumescent recipes. Moreover, their peculiar structure (Figure 3) accounts for the possibility of being exploited as reservoirs for entrapping flame retardants (very often phosphorus-based FRs) [32]. 

**Figure 4:**
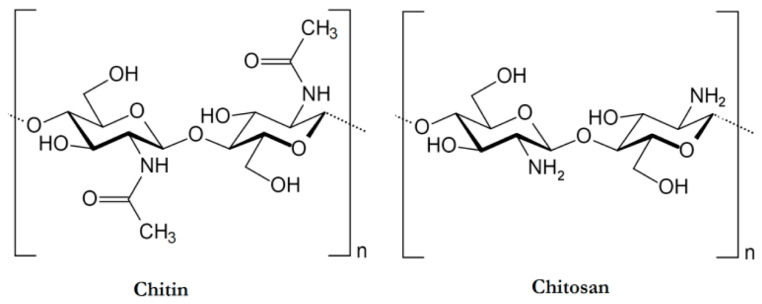
Chemical structure of chitin and chitosan. Reprinted from [34] under CC-BY license.

Chitosan can be obtained through partial or full deacetylation processes from chitin (poly (β-(1 → 4)-*N*-acetyl-d-glucosamine)), the second most abundant natural polysaccharide that is present in the exoskeleton of arthropods, in the cell wall of yeasts and fungi, as well as in the shells of crustaceans. The chemical structures of chitin and chitosan are schematized in Figure 4.

Again, the use of chitosan in flame retardant applications relies on its carbohydrate structure, which can be exploited as an effective carbon source. Moreover, the chemical modification of chitosan allows for its direct utilization as an efficient flame retardant for different polymer systems, such as thermoplastic polyurethane (chitosan crosslinked with bis-(4-formylphenyl)-phenyl-phosphonate [35], phosphorylated and combined with montmorillonite nanosheets [36], or even reacted with toluene diisocyanate and microencapsulating melamine polyphosphate [37]), and linear low-density polyethylene (using a melamine salt of chitosan phosphate [38]).

Tartaric acid is a sugar-derived acid that is obtained as a by-product from the wine industry during the fermentation processes to which grape stocks are subjected. The conversion of tartaric acid previously treated with phosphorus-containing co-reactants into the resultant diethyl ester structure allows for the design of effective flame retardants that exhibit a remarkable charring effect [39]. 

**Figure 5:**
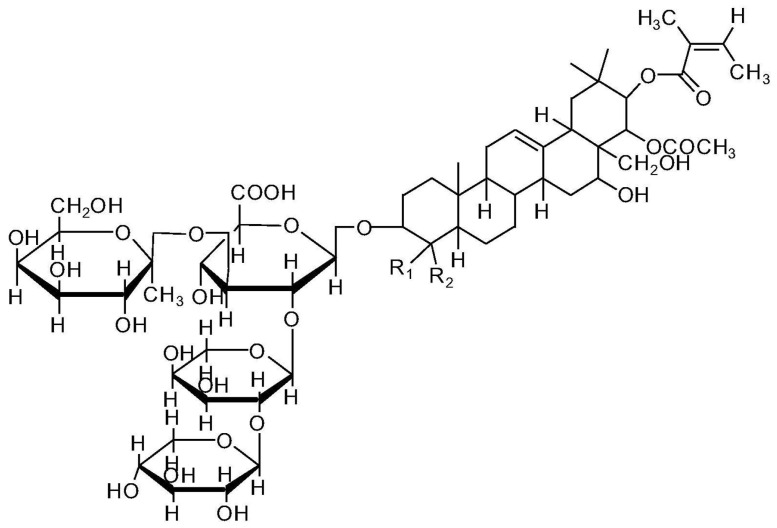
Chemical structure of tea saponin. Reprinted with permission from [43]. Copyright Elsevier, 2019.

Tea saponin, a natural carbohydrate, can be recovered from camellia seeds and consists of saccharide portions combined with a triterpene-derived molecule (Figure 5). Apart from its standard use as a surfactant in food applications [40], tea saponin can be exploited as both a blowing agent and a carbon source in flame retardant formulations [41,42].

### 2.2. Nucleic Acids

**Figure 6:**
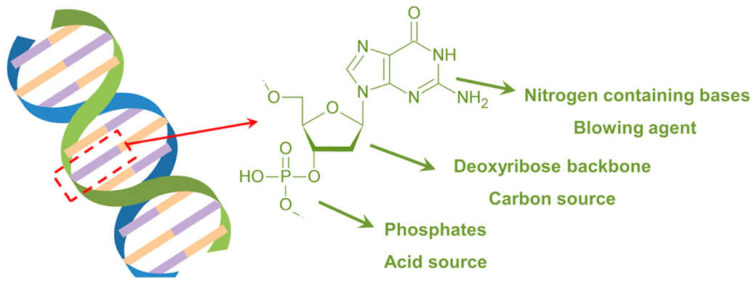
Structure of DNA. Adapted with permission from [45]. Copyright American Chemical Society, 2016.

DNA (deoxyribonucleic acid) and RNA (ribonucleic acid) are nucleic acids carrying the genetic information that is read in cells to make the RNA and proteins, by which living things can function [44]. There are some differences between DNA and RNA: in fact, DNA is double-stranded, forming a double helix (Figure 6), while RNA is usually single-stranded. Further, the sugar in DNA is deoxyribose, whereas RNA contains ribose. Finally, DNA utilizes the bases adenine, thymine, cytosine, and guanine, while RNA uses adenine, uracil, cytosine, and guanine.

**Figure 7:**
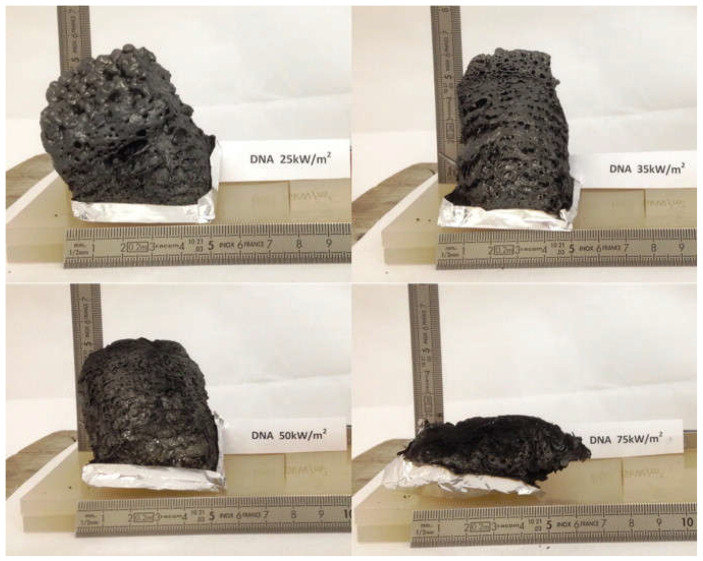
Intumescent features of DNA, as assessed through cone calorimetry tests performed at different irradiative heat fluxes (from 25 to 75 kW/m^2^). Reprinted with permission from [46]. Copyright Elsevier, 2014.

Regardless of their specific chemical structure, nucleic acids undoubtedly behave as all-in-one intumescent flame retardants, as they contain an acid source (phosphate groups), a blowing agent (the N-containing bases), and a carbon source (deoxyribose or ribose units). The remarkable intumescent character was documented through cone calorimetry tests carried out at different irradiative heat fluxes (Figure 7). 

Several papers well describe the effectiveness and suitability of nucleic acids as FRs for bulk polymers, foams, and fabrics; these biomacromolecules show a prevalent flame retardant effect in the condensed phase [47,48,49].

### 2.3. Protein-Based FRs

The use of such proteins as whey proteins, caseins, and hydrophobins as quite effective flame retardants has been somehow limited to cellulosic (cotton) fabrics. The ability of the main components of whey proteins (i.e., α-lactalbumin and β-lactoglobulin), recovered from a waste stream of the cheese industry, to form edible and biodegradable films has extensively been investigated [50]. Moreover, their char-forming character (with a consequent decrease in the burning rate in horizontal flame spread tests) was demonstrated on cotton fabrics [51].

Caseins are phosphorus-containing proteins recovered as a co-product of the milk industry [52]. They exhibit char-forming features when deposited on cotton, polyesters, or their blends, in particular the char that originates during the application of the flame behaves as a protective layer, limiting the degradation of the underlying fabric material [53,54].

Hydrophobins are produced by filamentous fungi; they consist of small cysteine-rich proteins (molecular weight of about 10 kDa) containing S and N atoms, organized as a small β-structured core formed by eight conserved cysteine residues that create four disulfide bridges [55]. As regards the flame-retardant features of these proteins, the decomposition of their disulfide bonds favors the release of acidic species (sulfuric acid), responsible for the occurrence of dehydration reactions and the formation of a stable and protective char layer [56,57]. 

### 2.4. Vegetable Oil-Based FRs

Vegetable oils mainly consist of triglycerides, i.e., esters of glycerol with three fatty acids with 10–22 carbon atoms; they can be recovered from oilseeds [58]. Regarding flame retardancy, the hydroxyl functionalities of vegetable oils can be exploited for obtaining phosphorus-containing ester groups (i.e., phosphorylated polyols), mainly suitable for the design of fireproof polyurethane foams [59,60].

### 2.5. Bio-Sourced Aromatic FRs

**Figure 8:**
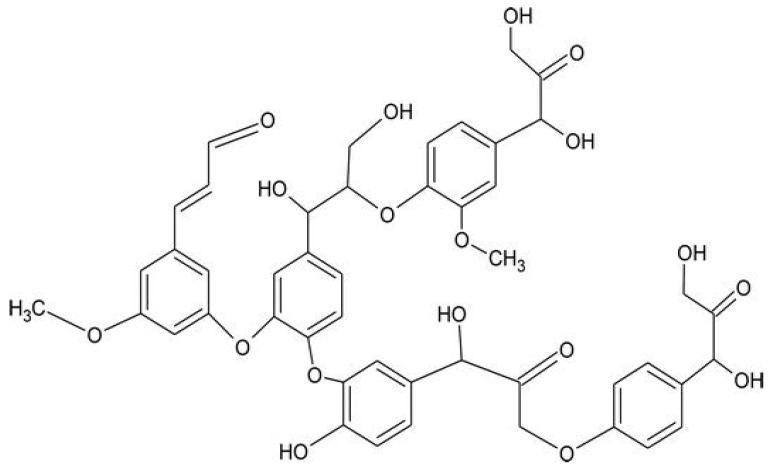
Chemical structure of lignin. Reprinted from [63] under CC-BY license.

Lignin (Figure 8) is the second-most abundant carbohydrate resource after cellulose and a key source of natural aromatic building blocks [61]. The yearly worldwide production of lignin is about 50 million tons, originating from the byproducts of the pulp industry [62]. The structure of lignin is very complex and variable, as it consists of a crosslinked, three-dimensional network originating from phenylpropanoid units. 

Several functional groups are present in lignin, ranging from aldehydic and carboxylic groups to phenolic hydroxyl and methoxyl functionalities, also based on the plant species from which lignin is extracted [64]. In flame retardant applications, incorporating lignin in different polymers promotes a significant charring behavior (in other words, lignin acts as a carbon source in the condensed phase); the formed stable char slows down the diffusion of heat and oxygen, inhibiting the generation of gaseous species originating from the degradation process.

Moreover, the scientific literature well documents the possibility of combining lignin with other flame retardants, like phosphorus-containing additives or metal hydroxides [65], hence further increasing the charring effect provided by the synergism that often occurs between the components of the flame retardant recipe [66]. A further possibility is to chemically modify lignin, obtaining derivatives that exhibit flame-retardant characteristics. To this end, lignin can undergo phosphorylation reactions with chloro-phosphorus compounds or can react with phosphoric acid or phosphonate [67,68,69].

Finally, another possibility refers to the modification of lignin with nitrogen-containing compounds in the presence of phosphorus compounds. As a result, the so-modified lignin acts as an intumescent flame retardant, where lignin is the carbon source and nitrogen accounts for the swelling of the formed char, whose formation is catalyzed by the phosphorus compound (acid source) [70].

**Figure 9:**
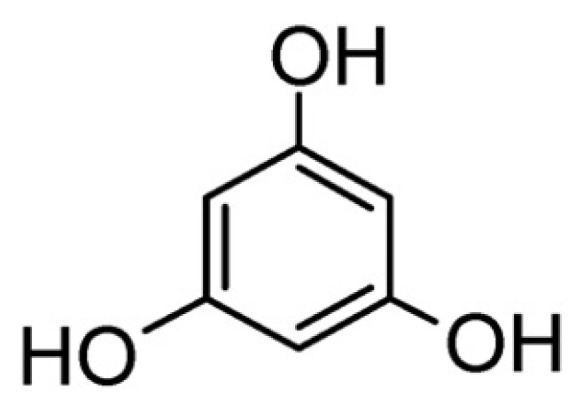
Chemical structure of phloroglucinol.

Phloroglucinol (Figure 9) is a phenolic molecule that can be exploited as a flame retardant for epoxy resins after phosphorylation, being prevalently active in the condensed phase [71].

**Figure 10:**
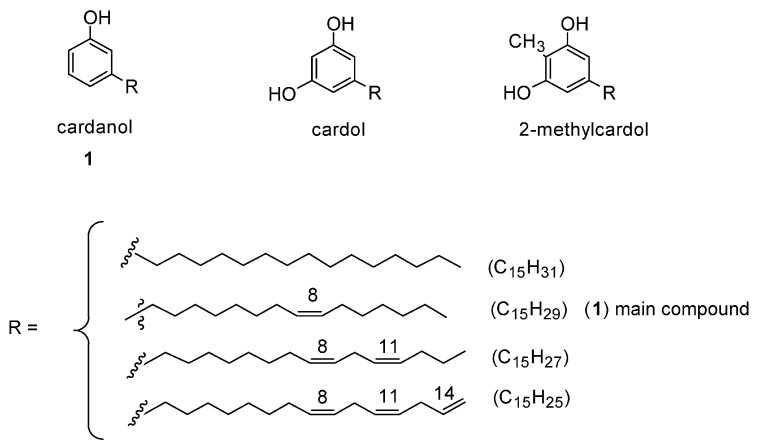
Chemical structures of cardanol, cardol, and 2-methylcardol, the constituents of technical-grade cashew nutshell liquid. Reprinted from [73] under CC-BY License.

Cardanol is the main constituent (about 84%), together with cardol and 2-methylcardol (Figure 10), of cashew nutshell liquid obtained from the spongy mesocarp of cashew nutshells. An interesting paper reports on the use of this bio-sourced product for the design of effective flame retardants for epoxy resins [72]. To this end, cardanol was first converted into a surfactant (by reaction with butane sulfone) and then exploited for the modification of layered double hydroxide. 

**Figure 11:**
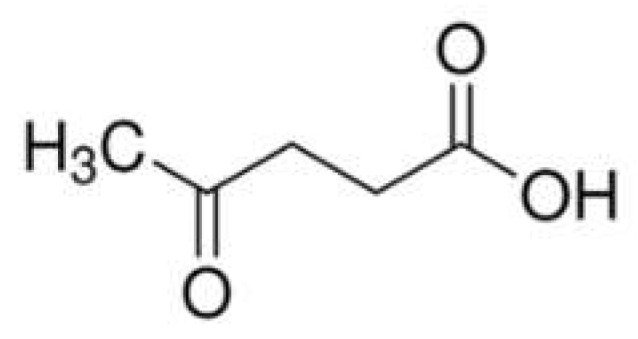
Chemical structure of levulinic acid (4-oxopentanoic acid).

Levulinic acid (4-oxopentanoic acid, Figure 11) is a keto acid that can be extracted from different biomasses; the concurrent presence of a carbonyl and carboxyl group makes this bio-sourced compound very attractive as a building block (i.e., as an intermediate) for the synthesis of different chemicals [74]. In particular, it can be converted into diphenolic acid that, in turn, can be employed for the synthesis of a phenolic flame retardant containing phosphorus and nitrogen elements, suitable as a charring agent in the condensed phase [75].

### 2.6. Phytic Acid and Phytates

**Figure 12:**
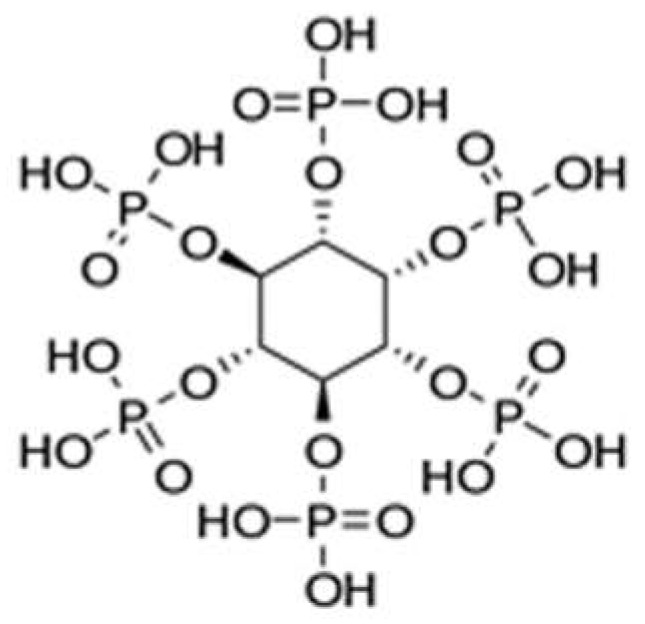
Chemical structure of phytic acid. Reprinted from [83] under CC-BY License.

Phytic acid (Figure 12) and its salts (aluminum, lanthanum, iron, and sodium phytates, among a few to mention) represent perhaps the most recent “discovery” in the field of bio-sourced flame retardants. Phytic acid can be extracted from several sustainable resources such as cereal grains, beans, and oilseeds. Moreover, the high phosphorus content (i.e., 28 wt.% based on molecular weight), structured in the form of six phosphate groups, accounts for the rising interest that this biomolecule gathered in the last ten years, for different application sectors (as an antioxidant, corrosion inhibitor, and anticancer additive, among a few to mention [76,77]). As will be described in the next paragraphs, phytic acid is a very efficient char-former, further contributing to providing excellent flame retardant features when in combination with other carbon sources (like chitosan [78,79]) or with nitrogen-containing additives (such as melamine, urea, or polyethyleneimine [80,81,82], for the design of effective intumescent systems. 

Table 1 summarizes the main fire-retardant mechanisms promoted by the different classes of bio-sourced products applied to textile materials. Generally speaking, their FR action involves the condensed phase, with the formation of a stable, aromatic, and protective char, usually through dehydration reactions that take place during the decomposition/activation of the bio-based flame retardant additive. Further, some of the biobased FRs can be active in the gas phase through dilution effects (i.e., by diluting the oxidizable gaseous species, which fuel the flame, with inert gases generated by the decomposition/activation of the bio-based FRs) or by exerting a “scavenging action,” resulting in the entrapment of the reactive free radicals that are responsible for the flame propagation [84]. 

## 3. Bio-Sourced Flame Retardants for Textiles: Recent Outcomes

This paragraph will summarize the very recent and most interesting results (within 2021 and 2024 years) of the use of bio-sourced flame retardants for conferring fireproof characteristics to various textile materials. The different systems will be classified based on the type of textile material employed (namely, referring to cotton, wool, and silk, which are currently most investigated, but also providing some recent examples of other fabrics and blended fabrics).

Table 2 collects the investigated bio-based flame-retardant systems for textiles and the main research outcomes. It is noteworthy to underline that all these bio-sourced products can significantly contribute to human safety, as they are able to stop (or at least remarkably slow down) the propagation of a flame and account for a decreased heat release and heat release rate. Moreover, in some cases, they suppress the smoke generation during a fire accident, thus allowing people to escape from the places where a fire is occurring. Finally, they may ensure durability (i.e., washing speed), which is often mandatory for several applications, to prolong the effectiveness of the FR treatments. 

### 3.1. Cotton Fabrics

Liao and co-workers [85] synthesized a bio-based reactive flame-retardant ammonium salt of arginine hexamethylenephosphonic acid, grafted onto cotton at three different dry add-ons (about 15, 19, and 22 wt.%.). As assessed by cone calorimetry tests (irradiative heat flux: 35 kW/m^2^), the fabrics with the highest flame-retardant add-on did not ignite; moreover, they achieved a Limiting Oxygen Index as high as 45.1% and self-extinction in vertical flame spread tests. These findings were ascribed to the extended charring effect provided by the bio-sourced salt: upon the application of the flame or the exposure to the irradiative heat flux, the decomposition of the flame-retardant accounted for the formation of phosphoric acid or polyphosphoric acid, able to catalyze the dehydration of the cotton substrate toward the development of a stable aromatic char. Thanks to the establishment of P-O-C covalent bonds, the treated fabrics (containing 19 or 22 wt.% of the bio-sourced salt) could resist 50 laundry cycles, hence showing remarkable washing speed.

**Figure 13:**
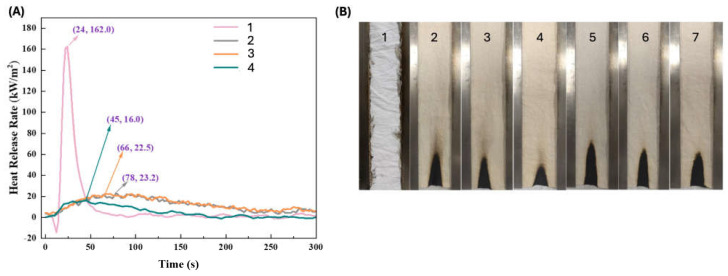
Results from vertical flame spread tests (**A**) and forced combustion tests performed at 35 kW/m^2^ irradiative heat flux (**B**) for cotton and the fabrics treated with ammonium starch phosphate (ASP) at different loadings, before and after 50 laundry cycles. Legend: 1 = untreated cotton; 2, 3, 4 = cotton treated with ammonium starch phosphate at different loadings (24.1, 26.9, and 33.1 wt.%, respectively); 5, 6, 7 = samples 2, 3 and 4 tested after 50 laundry cycles. Adapted with permission from [86]. Copyright Elsevier, 2022.

Ammonium starch phosphate, derived from biomass starch through the reaction with phosphoric acid and urea, was employed to confer durable and effective flame retardant features to cotton [86]. In particular, the cellulosic fabrics were treated with aqueous ammonium starch phosphate solutions, using dicyandiamide as a catalyst, and achieved 24.1, 26.9, and 33.1 wt.% final dry add-ons. All the treated fabrics reached self-extinction in vertical flame spread tests and did not ignite at 35 kW/m^2^ during forced-combustion tests (Figure 13). Moreover, all the treated fabrics showed high durability, as they were still self-extinguishing after 50 laundry cycles carried out according to the AATCC standard [110] (see samples 5–7 of Figure 13B). These findings were attributed to the formation of P-O-C covalent bonds between the bio-sourced flame retardant and the cotton fabric.

Ma and co-workers [87] exploited the layer-by-layer technique [111,112] to provide cotton fabrics with enhanced flame-retardant properties. To achieve this aim, 2 or 4 bi-layered assemblies made of synthesized graphite carbon nitride and phosphorylated chitosan were deposited on the cellulosic substrate through dipping. Four bi-layers accounted for self-extinction in vertical flame spread tests, the achievement of Limiting Oxygen Index values as high as 30.1%, and a significant decrease in the peak of Heat Release Rate (by about 56%) during forced combustion tests (carried out at 35 kW/m^2^ irradiative heat flux). Moreover, the photocatalytic activity of graphite carbon nitride allowed for interesting self-cleaning features, with 90% efficiency in the removal of an organic dye (i.e., Rhodamine B) within 15 min in the presence of just 2 bi-layers of the deposited assembly. 

**Figure 14:**
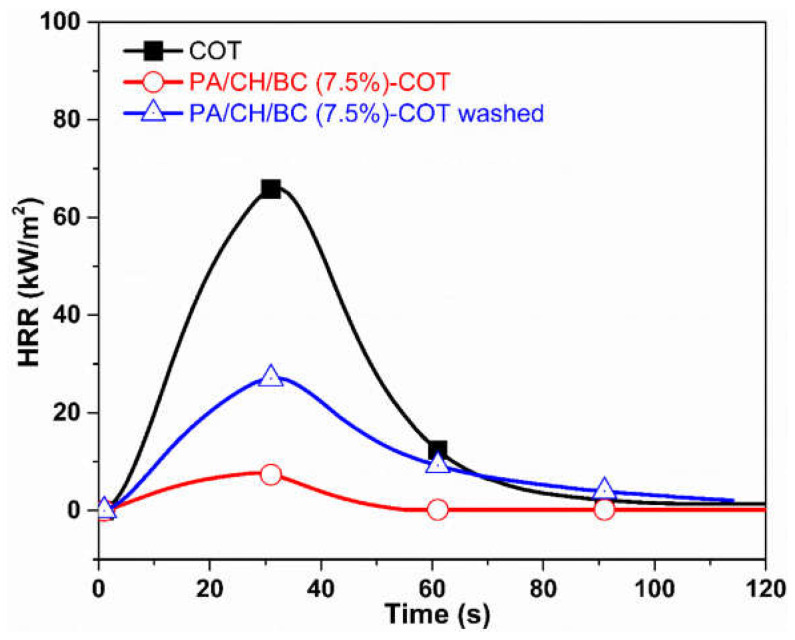
Results from forced combustion tests carried out at 35 kW/m^2^ on neat cotton (COT), cotton treated with 5 bilayers of chitosan/Biochar (7.5 wt.% concentration) and phytic acid (PA/CH/BC (7.5%)-COT), and on the treated fabric after 10 laundry cycles (PA/CH/BC (7.5%)-COT washed). Reprinted with permission from [79]. Copyright Elsevier, 2022.

Cheng and co-workers [79] demonstrated the effectiveness of the layer-by-layer deposition of 5 bilayers of chitosan/biochar (from aqueous solutions of chitosan at different biochar loadings, namely: 5, 7.5, and 10 wt.%) and phytic acid for conferring semi-durable flame retardant features to cotton fabrics. In particular, the assembly derived from the dispersion of chitosan/biochar at 7.5 wt.% of the carbon filler allowed for achieving 64.1% of Limiting Oxygen Index (vs. 18.6% for the untreated cellulosic substrate) and promoted a remarkable decrease in the peak of Heat Release Rate (by 88.3%) and Total Heat Release (−87%) compared to the control fabric. Finally, the proposed surface-engineered treatment exhibited an interesting washing fastness (to 10 laundry cycles), despite a certain worsening of the flame retardant performance (Figure 14).

A similar approach was then exploited, depositing a bi-layered assembly consisting of laccase and phytic acid (15.6 wt.% of the final dry add-on on cotton) [88]. The treated fabrics showed self-extinction in vertical flame spread tests, as well as a Limiting Oxygen Index of 43% (vs. 18.8% for the untreated fabric). Further, pyrolysis combustion flow calorimetry tests highlighted a remarkable decrease in both the peak of Heat Release Rate (from 310-untreated cotton to its 36 W/g-treated counterpart) and Total Heat Release (from 13.5 to 1.7 kJ/g), hence evidencing the high protection exerted by the deposited assembly. Finally, as assessed by thermogravimetric analyses coupled with FTIR spectroscopy and by Raman spectroscopy measurements carried out on the residues after flammability tests, the flame retardant assembly was active in both gas and condensed phases. 

**Figure 15:**
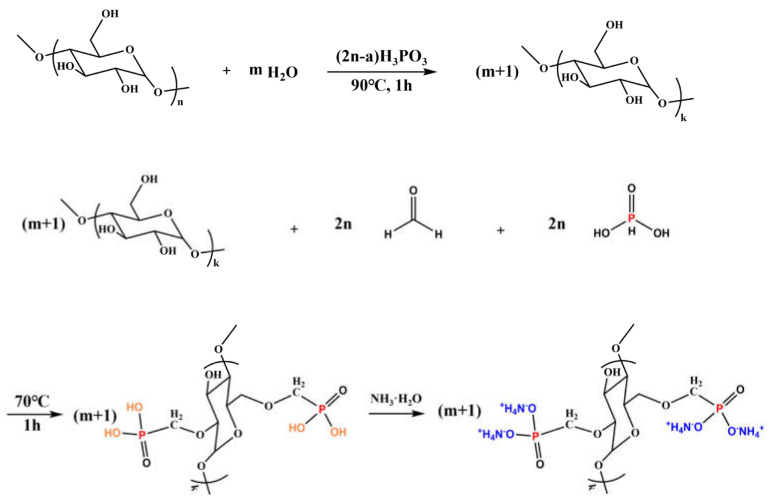
Synthesis of 2,6-dimethoxy polysaccharide ammonium phosphate. Reprinted with permission from [89]. Copyright Elsevier, 2023.

Lu and co-workers [89] treated cotton fabrics with 2,6-dimethoxy polysaccharide ammonium phosphate synthesized on purpose (Figure 15); in particular, three different weight gains were employed, namely 20, 25, and 30 wt.%. As revealed by vertical flame spread tests, all the treated fabrics achieved self-extinction, regardless of the flame retardant loading. Moreover, the Limiting Oxygen Index values were found to increase with increasing the FR loading, up to 49.4% (vs. 17.6% for the untreated cellulosic substrate). Further, the formulation containing 30 wt.% of 2,6-dimethoxy polysaccharide ammonium phosphate accounted for a remarkable decrease in the peak of Heat Release Rate (−93%) and Total Heat Release (−50%) compared with the untreated fabric. This finding was ascribed to the effectiveness of the flame retardant in the condensed phase, with the formation of a thermally stable and protective char. Conversely, the application of the bio-based flame retardant was responsible for a significant increase in smoke production, with a Total Smoke Release as high as 50.2 m^2^/m^2^ (vs. 2.3 m^2^/m^2^ for the untreated fabric). Finally, the formation of P-O-C covalent bonds between the flame retardant and the underlying cotton accounted for the high washing speed of the treated fabrics, which could resist 50 laundry cycles.

**Figure 16:**
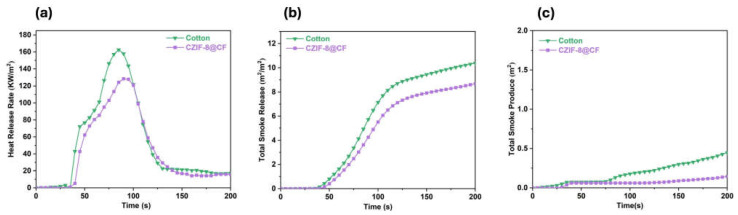
Heat release Rate (**a**), Total Smoke Release (**b**), and Total Smoke Production (**c**) curves of untreated cotton (cotton) and the fabric treated with the zeolitic imidazolate framework-8 modified with chitosan and Zn^2+^ (CZIF-8@CF). Adapted with permission from [90]. Copyright Elsevier, 2023.

Aiming to design multifunctional cotton fabrics, Cui and co-workers [90] in situ grew a zeolitic imidazolate framework-8 previously modified with chitosan and Zn^2+^. Apart from an important antibacterial activity against S. aureus and E. coli (with antibacterial rates as high as 99.9% for both) and substantial UV resistance, the treated fabrics exhibited a significant decrease in peak of Heat Release Rate, Total Heat Release, Total Smoke Release, and Total Smoke Production (respectively by 21.5, 16.4, 16.3, and 61%), as revealed by forced-combustion tests carried out at 35 kW/m^2^ irradiative heat flux (Figure 16). This finding was ascribed to the covalent bonding occurring between chitosan and a part of Zinc ions within the zeolitic imidazolate framework-8, which accounted for synergistic effects, as supported by the calculated synergistic effectiveness parameter (1.63) [113]. Finally, the proposed multifunctional treatment could resist 50 laundry cycles performed following the AATCC standard [110].

Very recently, Chen and co-workers [91] exploited the Schiff reaction of furfural and furfurylamine to synthesize a fully bio-derived phosphorylated furan-based flame retardant. The latter was applied to cotton and non-woven Poly(lactic acid) fabrics at three different loadings, namely 6, 12, and 16 wt.%. The fabrics containing at least 12 wt.% of the phosphorylated furan-based flame retardant were self-extinguishing in vertical flame spread tests. Moreover, compared to the untreated fabric, the presence of 16 wt.% of flame retardant loading accounted for a significant decrease in the peak of Heat Release Rate and Total Heat Release (respectively by about 71 and 38%) in forced-combustion tests (35 kW/m^2^ irradiative heat flux), highlighting the high char-forming character of the biobased flame retardant in the condensed phase.

A doctor-blading approach was successfully exploited for conferring flame-retardant features to cotton fabrics through the coating of a polyelectrolyte complex made of branched polyethylene imine (cationic component) and cellulose nanocrystals (anionic counterpart) [92]. The cellulosic substrate, treated on both sides with the polyelectrolyte complex (final weight gain: 10 wt.%), achieved self-extinction in horizontal flame spread tests. Moreover, SEM analyses carried out on the burnt material highlighted the intumescent character of the proposed treatment (with the formation of expanded micro-bubbles), which favored the formation of a stable and protective char (made of amorphous carbonaceous structures including polyaromatic clusters, as clearly pointed out through Raman spectroscopy measurements). Finally, the treated fabrics showed acceptable durability after leaching tests in mild conditions (i.e., immersing the treated fabrics in deionized water under stirring at 23 °C for 10 min).

**Figure 17:**
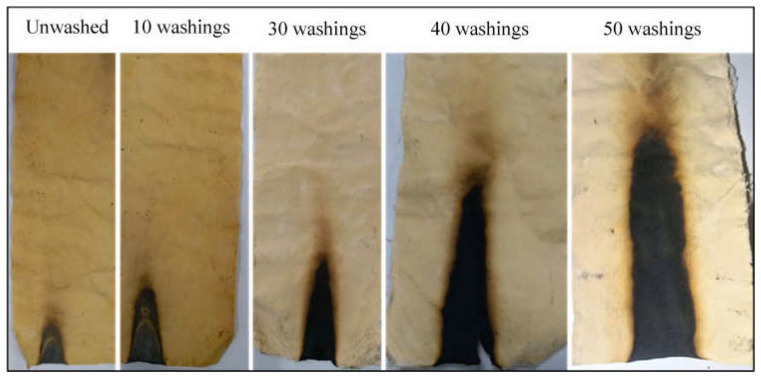
Results from vertical flame spread tests performed on cotton fabrics treated with a 3-(2-aminoethylamino)-propyltrimethoxysilane sol containing 14 wt.% phytic acid, after different laundry cycles. Reprinted with permission from [93]. Copyright Elsevier, 2024.

Safdar and co-workers [93] proposed a bio-based flame retardant treatment for cotton fabrics, employing phytic acid previously incorporated into 3-(2-aminoethylamino)-propyltrimethoxysilane sol. A total of 14 wt.% of phytic acid in the sol provided self-extinction to the treated cotton; furthermore, as shown in Figure 17, the flame-retarded fabrics were still self-extinguishing after 50 laundry cycles carried out according to the ISO 105-C10 standard test method [114].

Very recently, five bi-layers of egg white proteins (positively charged) and magnesium lignosulfonate–diammonium phosphate (negatively charged) were layer-by-layer deposited on cotton fabrics, achieving 6.8 wt.% of dry add-on [94]. The resulting fabrics reached self-extinction in both vertical and horizontal flame spread tests. Further, pyrolysis-combustion flow calorimetry analyses highlighted a remarkable decrease in the peak of Heat Release Rate (−60.1%), Total Heat Release (−59.3%), and Fire Growth Capacity (−81.5%), hence indicating the effective char-forming action of the deposited assembly in the condensed phase. Finally, the layer-by-layer architecture did not impact the mechanical features of cotton and its air permeability.

Li and co-workers [95] designed a flame retardant mixture for cotton fabrics, made of lignin-silica-based liquid (extracted from rice husk) and 9, 10-dihydro-9-oxa-10-phosphaphenanthrene-10-oxide. This latter was added at different loadings (namely 5, 10, and 15 g) to 100 mL of the lignin-silica-based liquid; then, cotton fabrics were immersed into the resulting FR solutions, achieving dry add-ons between 23.2 and 27.3 wt.%. As assessed by vertical flame spread tests, all the treated fabrics, irrespective of the FR loading, achieved self-extinction. Moreover, pyrolysis-combustion flow calorimetry tests highlighted a significant decrease in both peak of Heat Release Rate (by about 78%) and Total Heat release (by around 65%), as compared to the control fabric. All these findings were attributed to the char-forming character of the bio-based flame retardant.

A multifunctional (i.e., flame retardant and antibacterial) treatment was recently proposed by Huang and co-workers [96], who treated cotton with chitosan protonated with amino trimethylene phosphonic acid at different loadings (namely, 5.5, 8.5, and 11.5 wt.%). Self-extinction in vertical flame spread tests was achieved only for the fabrics treated with the highest FR loading. Moreover, 11.5 wt.% of the flame retardant accounted for Limiting Oxygen Index values as high as 29.7%. Further, forced combustion tests (irradiative heat flux: 35 kW/m^2^) revealed a remarkable decrease in peak of Heat Release Rate (−87%), Total Heat Release (−62%), and Total Smoke Release (−50%) as compared to the untreated cellulosic substrate. Moreover, the deposited coatings accounted for an important antibacterial activity, with 95.1 and 99.9% antibacterial rates against *E. coli* and *S. aureus*, respectively. Finally, the proposed multifunctional treatment did not affect either the whiteness or breathability of the fabrics.

Liu and co-workers [97] exploited the sol-gel technology for depositing a coating based on γ-ureidopropyltriethoxysilane and ammonia phytate. 9.9 wt.% final dry add-on accounted for self-extinction in vertical flame spread tests and 31% Limiting Oxygen Index values (vs. 18% for the control fabric). Moreover, compared to the untreated cotton, a significant decrease in peak of Heat Release Rate (by 48%), Total Heat Release (by 40%), and Total Smoke Production (by 48%) was observed in forced-combustion tests (carried out under 35 kW/m^2^ irradiative heat flux). These findings were ascribed to the high char-forming character of the deposited coating, which facilitated the dehydration and carbonization of the cellulosic substrate, favoring the formation of a stable and protective carbonaceous residue. 

### 3.2. Wool Fabrics

**Figure 18:**
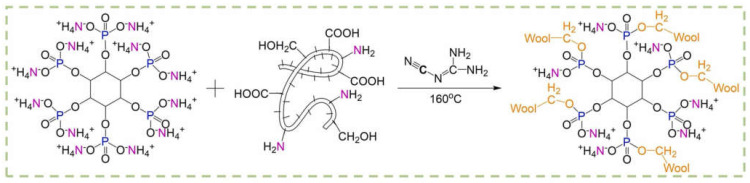
Grafting reaction of ammonium phytate onto wool fabrics. Reprinted with permission from [98]. Copyright Elsevier, 2022.

**Figure 19:**
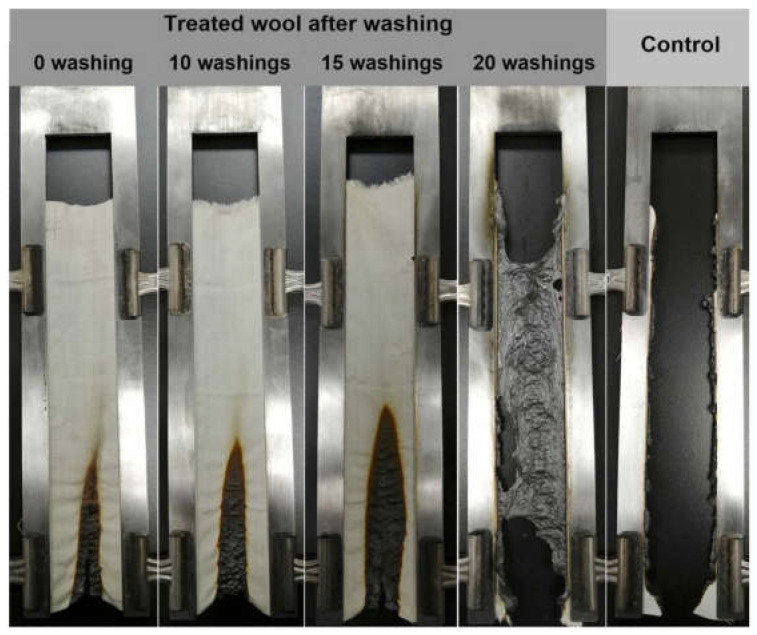
Results of vertical flame spread tests performed on wool fabrics treated with ammonium phytate (dry add-on: 14.2 wt.%) before and after laundry cycles. Reprinted with permission from [98]. Copyright Elsevier, 2022.

Cheng and co-workers [98] proposed a durable and bio-based flame-retardant treatment for wool fabrics, exploiting the covalent grafting of ammonium phytate in the presence of dicyandiamide, employed as a catalyst (Figure 18). As assessed during vertical flame spread tests, the treated fabrics (ammonium phytate dry add-on beyond 7.5 wt.%) achieved self-extinction. Moreover, pyrolysis-combustion flow calorimetry tests highlighted a significant decrease in the peak of Heat Release Rate (by about 34.1%) compared with the untreated textile. These findings were ascribed to the high char-forming character of the deposited bio-based flame retardant that, upon decomposition, favored the dehydration of the protein substrate, giving rise to the formation of a stable and protective char. Finally, the fireproof features were also maintained after 15 laundry cycles according to the AATCC standard [110] (Figure 19).

### 3.3. Silk Fabrics

Professor Guan’s group has performed most of the recent research efforts on the design and application of bio-based flame retardants on silk fabrics. They will be summarized in the following. 

**Figure 20:**
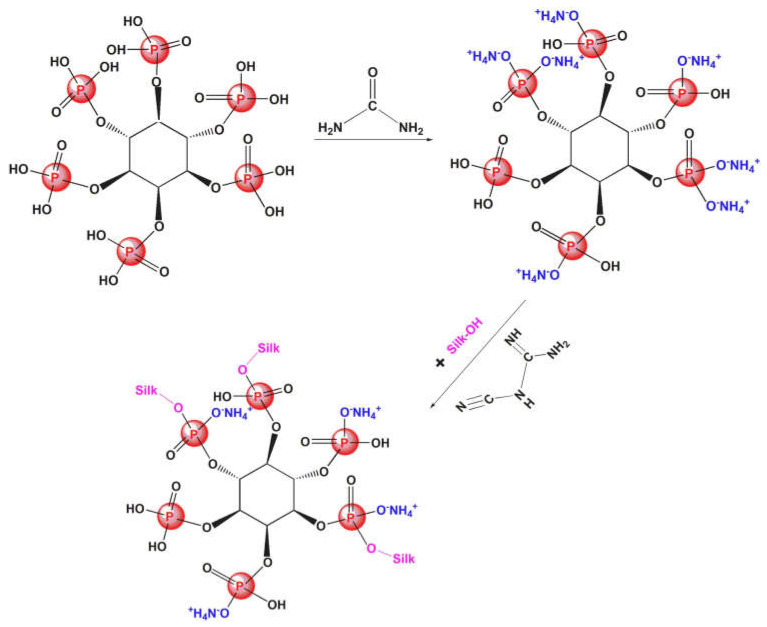
Synthesis of phytate urea salt and its covalent crosslinking with silk fabrics. Adapted with permission from [99]. Copyright Elsevier, 2021.

A pad-dry-cure technique was exploited by Huang and co-workers [99] to coat silk fabrics with phytate urea salt, a reactive bio-based flame retardant derived from the reaction between phytic acid and urea (Figure 20). Different weight gains of the coated fabrics, ranging from 3 to 23 wt.%, were investigated. All the designed coatings accounted for self-extinction in vertical flame spread tests. Moreover, 23 wt.% FR loading was responsible for a significant lowering of the peak of Heat Release Rate (−41%) and the Total Heat release (−59%), compared to untreated silk. Finally, the capability of the reactive flame retardant to covalently bond to the silk substrate resulted in excellent washing speed of the treated fabrics (up to 35 laundry cycles). 

**Figure 21:**
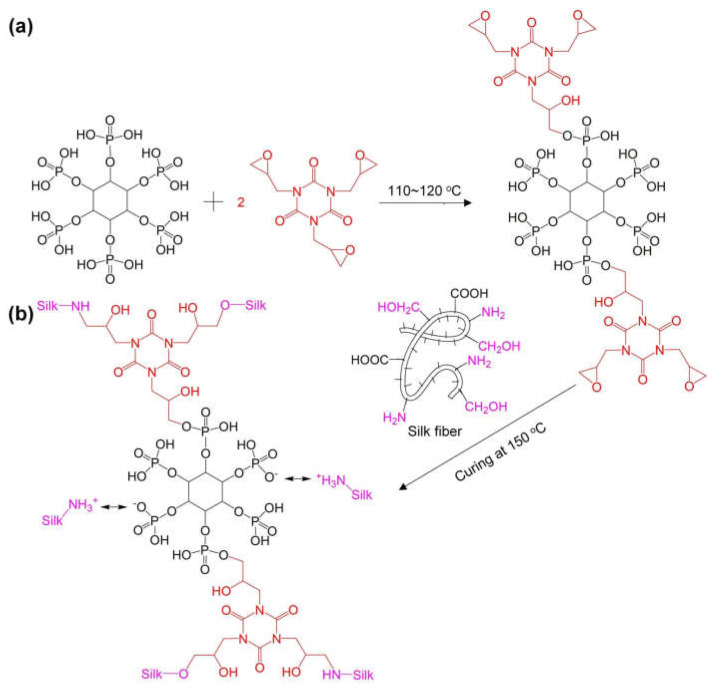
Synthesis of glycidyl phytate isocyanurate (**a**) and its covalent crosslinking with silk fabrics (**b**). Reprinted with permission from [100]. Copyright Elsevier, 2023.

Cheng and co-workers [100] exploited the ring-opening reaction of 1,3,5-triglycidyl isocyanurate with phytic acid, hence obtaining glycidyl phytate isocyanurate, which was crosslinked with silk fabrics (Figure 21). A total of 13.6 wt.% of the flame retardant ensured self-extinction in vertical flame spread tests and a Limiting Oxygen Index of 32.5%. Further, as assessed through pyrolysis-combustion flow calorimetry tests, the proposed flame retardant treatment accounted for an important decrease in the peak of Heat Release Rate, Total Heat Release, and Heat Release Capacity (respectively by 46.1, 29.6, and 39.6%) compared with the untreated fabric. Glycidyl phytate isocyanurate was proven to act as an effective intumescent flame retardant in both the condensed and gas phases. In fact, the phosphate groups behaved as the acid source, the triazine ring acted as the blowing agent (generating inert gaseous isocyanate fragments that exerted a gas phase dilution mechanism), and the glycidyl groups, together with the silk fabric, played as the carbon source. Finally, the occurrence of the crosslinking reaction between glycidyl phytate isocyanurate and silk was responsible for the washing fastness of the flame-retarded fabrics (up to 30 laundry cycles according to the AATCC standard [110]. 

Pursuing this research, the same group [101] synthesized pentaerythritol phytate ethylenediaminetetraacetic ester through the esterification of phytic acid, pentaerythritol, and ethylenediaminetetraacetic acid; two different dry add-ons were selected, namely 10.7 and 13.5 wt.%. The proposed treatment was very effective in providing silk fabrics with flame retardant features: in particular, all the treated fabrics, regardless of the FR loading, were self-extinguishing in vertical flame spread tests. Moreover, Limiting Oxygen Index values increased with increasing the dry add-on, moving from 23.6% (untreated silk) to 32.8 and 35.2% (treated fabrics). Also, pyrolysis-combustion flow calorimetry tests highlighted the effectiveness of the protection exerted by the bio-based flame retardant on the underlying fabrics: 13.5% FR loading accounted for a remarkable decrease in the peak of Heat Release Rate (by about 41%) and Total Heat Release (by about 49%), hence supporting the formation of a stable and protective char during the pyrolysis of the fabric in the condensed phase. Interestingly, the flame-retarded fabrics could withstand at least 15 laundry cycles (washing the fabrics at 40 °C for 30 min in the presence of 2 g/L of detergent, 50:1 liquor ratio). 

**Figure 22:**
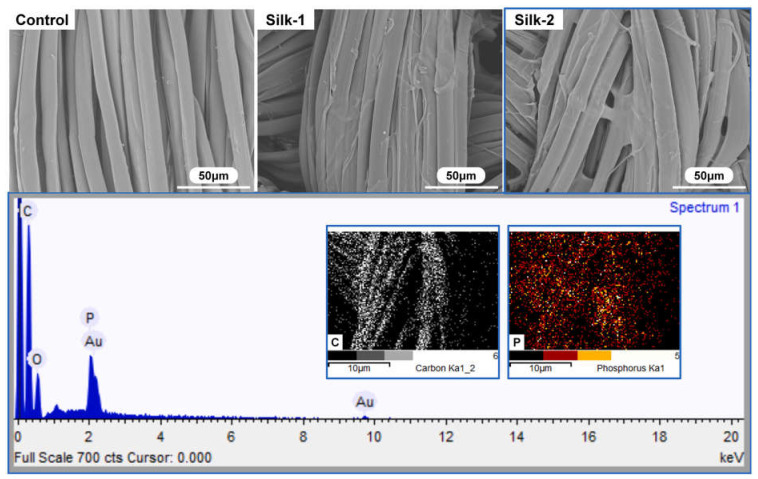
Typical SEM micrographs and EDS analysis of coated silk samples. Legend: Control = untreated silk; Silk-1 = the fabric treated with the reactive bio-based FR (11.8% dry add-on); Silk-2 = the fabric treated with the reactive bio-based FR (14.2% dry add-on). Reprinted with permission from [102]. Copyright Elsevier, 2023.

In a further research effort, Cheng and co-workers [102] synthesized a bio-based, reactive, and intumescent reactive flame-retardant coating for silk using phytic acid, triethanolamine, and citric acid, at two different loadings, namely 11.8 and 14.2%. The chemical composition of the FR coatings and their homogeneous coverage of the fabrics (Figure 22) accounted for an important protection action, witnessed by the attainment of self-extinction in vertical flame spread tests, regardless of the FR loading, and by the high Limiting Oxygen Index values achieved (31 and 32.5%, vs. 23.5% for the pristine fabric). Further, pyrolysis-combustion flow calorimetry tests highlighted the FR effectiveness of the deposited coatings, significantly lowering both peak of Heat Release Rate (−43%) and Total Heat release (−57%). Because of the covalent grafting of the bio-based flame retardant onto the silk surface, the proposed FR treatment was durable and resistant to 30 washing cycles.

### 3.4. Other Fabrics

The possibility of conferring interesting flame retardant features to polyester fabrics by using a bio-based flame retardant was demonstrated by Zhang and co-workers [103]. To this end, the fabrics were dipped in a sodium alginate solution and subsequently complexed with Fe^3+^ (immersing the fabrics in a FeCl_3_ aqueous solution); the final dry add-ons were 23 and 42 wt.%. All the treated fabrics achieved V-0 classification in vertical flame spread tests; further, the highest FR loading accounted for a Limiting Oxygen Index value as high as 29% (vs. 20% for the untreated polyester fabric). Finally, forced-combustion tests carried out at 35 kW/m^2^ irradiative heat flux highlighted a significant decrease in the peak of Heat Release Rate (−70% for the polyester loaded with 42 wt.% of FR), Total Heat release (−12%), and Total Smoke Production (−27%) compared to the untreated fabric. All these findings were ascribed to the intense char-forming activity exerted by the bio-based flame retardant in the condensed phase.

**Figure 23:**
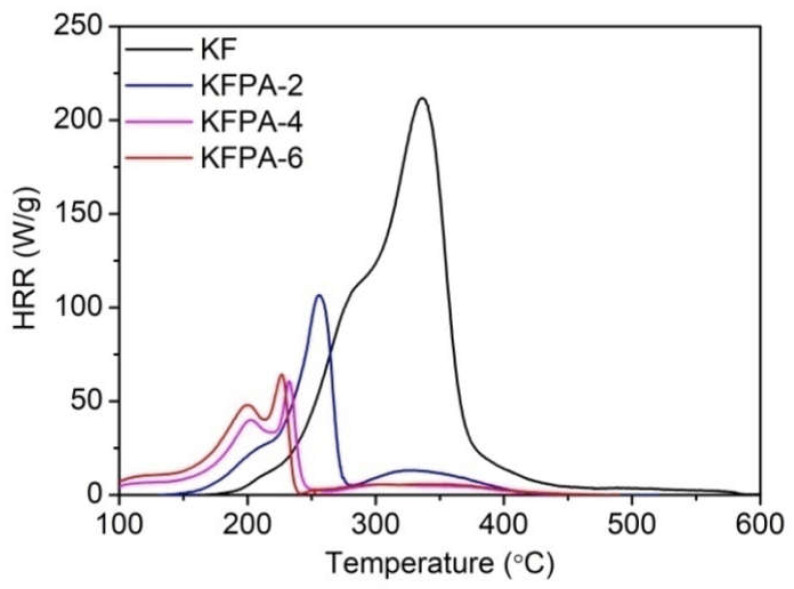
Heat Release Rate (HRR) vs. temperature curves for pristine kapok (KF) and the phosphorylated fabrics (KFPA-X, where X represents the phytic acid dosage in g). Reprinted from [105] under CC-BY License.

Jang and Tang [105] phosphorylated kapok fabrics with phytic acid in the presence of urea. Three different dry add-ons were considered, namely 3.36, 5.56, and 6.23%, which correspond to the phytic acid dosage (2, 4, and 6 g, respectively). All the treated fabrics reached self-extinction in vertical flame spread tests. Moreover, as assessed by pyrolysis-combustion flow calorimetry (Figure 23), at the highest loading, the bio-based FR remarkably decreased the peak of Heat Release Rate (by 69.6%), the Total Heat Release (by 78.4%) and the Heat Release Capacity (by 71.7%), further supporting its charring ability in the condensed phase. Finally, the so-obtained flame retarded fabrics exhibited good resistance to 50 laundry cycles, thanks to the grafting of phytic acid onto the cellulosic substrate through the formation of phosphate ester bonds.

Smith and co-workers [106] utilized the layer-by-layer method for depositing 15 quad-layers made of chitosan, phytic acid, and tannic acid on the surface of nylon-cotton blended fabrics. By exploiting the synergism taking place among the three components of the assembly, the treated fabrics (16.6 dry add-on) achieved self-extinction in vertical flame spread tests. Further, as assessed by pyrolysis-combustion flow calorimetry, these flame-retarded fabrics exhibited a decreased peak of Heat Release Rate (by 51%) and Total Heat Release (by 39%) compared to their control counterparts. Interestingly, the “hand” (i.e., the soft touch of the treated fabric) was not affected by the layer-by-layer assembly. 

Then, nylon-cotton blended fabrics were treated with coatings made of phytic acid and L-cysteine, employing an ion exchange method (final dry add-on about 30 wt.%) [107]. The proposed treatment accounted for a significant increase in Limiting Oxygen Index values, which shifted from 19.3 (control fabric) to 27.2%. Moreover, the treated fabrics achieved self-extinction in vertical flame spread tests and showed a decreased peak of Heat Release Rate (by 65.4%) and Total Heat Release (by 62.6%) compared to the untreated counterparts during forced-combustion tests (irradiative heat flux: 35 kW/m^2^). Further, the treatment accounted for outstanding smoke suppression, with a remarkable reduction in Total Smoke Production (−64.5%) and Smoke Production Rate (−67.6%). These findings were attributed not only to the char-forming ability of the treated fabrics in the condensed phase but also to the formation of incombustible gases (derived from the decomposition of L-cysteine), which exerted a dilution effect in the gas phase. Finally, the proposed bio-based coating could provide the treated fabrics with multifunctional features; indeed, after the flame retardant treatment, the fabrics also exhibited improved hydrophilicity and enhanced antibacterial properties against *E. coli* and *S. aureus*. 

Dong and co-workers [108] surface-modified a polyester/cotton blended fabric with a phytic acid-urea salt coating synthesized on purpose. A total of 12.3% of FR weight gain accounted for a Limiting Oxygen Index as high as 27.3% (vs. 17.1% for the pristine fabric) and self-extinction in vertical flame spread tests, without after-flame or after-glow phenomena. Moreover, as assessed by pyrolysis-combustion flow calorimetry tests, the treated fabrics (17.9 weight gain) showed decreased values of the peak of Heat Release Rate (by 42%) and Total Heat Release (by 39%), compared to the control fabric. These findings were attributed to the FR action in the condensed phase, with a significant formation of a stable and coherent char, exploiting the synergistic effects provided by phosphorus and nitrogen elements. Unfortunately, the proposed bio-based FR treatment exhibited poor washing fastness, thus limiting its potential applications. 

Very recently, Sun and co-workers [104] demonstrated the effectiveness of a coating made of phosphite, pentamethyldisiloxane, urea, and sodium alginate (32.6% FR dry add-on) in providing self-extinction and antidripping features to polyester fabrics. In particular, the treatment allowed for achieving a Limiting Oxygen Index value of 35.3%and accounted for a decrease in the peak of Heat Release Rate (by 45%), Total Heat release (by 57%), and Total Smoke Production (by 48%) as compared to the untreated fabric. These outcomes were ascribed to a mixed action in both condensed (through the formation of a stable and protective char) and gas (because of the release of PO• radicals scavenging the reactive-free radicals (H• and OH•) propagating the combustion) phases. Finally, the proposed treatments were semi-durable, as the fireproof features were partially lost during laundry cycles.

In a further effort toward the design of multifunctional treatments, Ding and co-workers [109] exploited an impregnation method for depositing mixtures of MXene and bio-based carbon dots on cotton/lyocell-blend fabrics. As assessed by vertical flame spread tests, the treated cellulosic substrates were self-extinguishing and achieved Limiting Oxygen Index values as high as 36%. Further, forced-combustion tests highlighted a significant decrease in the peak of (Heat Release Rate (by 74%) and Total Heat Release (by 28%). These findings were attributed to the char-forming character of the deposited coatings, as well as to the release of non-flammable gaseous species (such as H_2_O and CO_2_), which were able to dilute the flammable volatiles, hence lowering the fire risk. Moreover, the treated fabrics showed high electrical conductivity values (up to 31.8 S m^−1^) and sensing properties. Interestingly, all these multifunctional characteristics were maintained after 20 laundry cycles, thus highlighting the durability of the proposed treatments. 

## 4. Current Limitations and Perspective Trends in the Use of Bio-Sourced Flame Retardants

Undoubtedly, textile fire retardancy is experiencing a new “fashion” that is strictly correlated with the demand for sustainability in materials, chemicals, and technology processes, as depicted by the up-to-date circular economy concept. Indeed, the seeking of potential alternatives to standard/chemical flame retardants has promoted (and is still promoting) some efforts by both the academic and industrial worlds toward the identification of new products with low environmental impact and higher sustainability, trying to maintain the overall flame retardant properties at a satisfactory level. 

In this context, several bio-sourced products have already been selected, tested, and further implemented as potential substitutes in textile flame retardancy. Surely, most of these novel bio-based FRs have highlighted a very high potential for improving the fire-retardant features of different types of textile materials. 

However, these bio-based products still have still some drawbacks that limit the spread of their use in textile flame retardancy. 

First, their Technology Readiness Level [115] is still low (around 3), and therefore they are currently fully suitable and ready for lab-scale applications only. 

Further, any envisaged upscaling to a pilot, pre-industrial, or industrial scale is strictly correlated not only with the availability and suitability of industrial plants for this specific textile finishing but also with the cost-effectiveness of the processes, which additionally involves the cost of the bio-sourced flame retardants. Indeed, some of these latter (even among the most promising FRs), such as phytic acid and nucleic acids, are still very expensive and therefore suitable only for lab-scale investigations. However, their supply costs would likely be reduced by implementing the recovery/extraction methods (in terms of extraction yields and purity levels), leading to large-scale, low-cost, and highly efficient processes.

In addition, from a specific technical point of view, many of these bio-sourced flame retardants are soluble in water; this peculiarity is surely an advantage in terms of using low-environmental impact finishing processes (i.e., water baths), but represents a big limitation as far as durability (i.e., resistance to laundry cycles) is considered. Indeed, for several technical applications, the washing fastness of flame-retarded textiles is mandatory. In this context, several efforts have been carried out so far to enhance the durability of the flame-retardant treatments. The most successful ones (also reported in the present review work) involve covalent bonding (i.e., grafting or cross-linking) of the bio-based FR additive to the underlying textile, exploiting the presence of reactive functionalities on both components. In any case, the new strategies for conferring durability must be based on the exploitation of sustainable and low-environmental-impact approaches, hence limiting the use of strong chemicals.

Finally, notwithstanding the high flame retardant effectiveness exhibited by some bio-sourced flame retardants, particular attention must be devoted to the tuning of the “hand” (i.e., soft touch, comfort) of the treated textiles, avoiding an excessive increase in the fabric’s stiffness, which may limit the handling or even the wearability of the textile material itself.

## 5. Conclusions

The present review has tried to demonstrate the potential of bio-sourced products as effective flame retardants for different textile materials. The use of these bio-sourced products in flame retardancy well matches the existing needs for sustainability and low environmental impact, which are substantially circumscribed within the current circular economy concept. 

Undoubtedly, the odd, unpredictable, and even unreasonable idea (as it was 10 to 15 years ago) of exploiting bio-sourced flame retardant products significantly changed during the last decade, also thanks to the strict directives issued by the EU and USA, which are progressively limiting the utilization of some standard additives (mainly based on halogenated-brominated-products) for flame-retardant purposes because of toxicity and even carcinogenicity issues.

Further, the combination of a green, effective, and reliable chemistry revolving around bio-sourced flame retardants with the possibility of their extraction/recovery from wastes, crops, or by-products coming from other process chains (such as the food chain) may favor better exploitation of the currently limited resources at our disposal. 

In conclusion, notwithstanding the present limitations, some progress in the design, development, and implementation of novel bio-sourced flame retardants is expected for the forthcoming years, further strengthening the concept of circular economy and sustainability and allowing for wider exploitation of these bio-based products.

## Figures and Tables

**Table 1:** Main fire-retardant mechanisms of the bio-sourced FRs applied to textiles.

Type of Bio-Sourced Flame Retardant	Fire-Retardant Mechanism(s)
Cellulose	Carbon source Char-former after chemical modification
Hemicellulose	Carbon source
Starch	Carbon source Char-former after oxidation (i.e., conversion in polyoxoacids)
Isosorbide	Carbon source Char-former after chemical modification
Cyclodextrins	Carbon source in intumescent systems
Chitosan	Carbon source Char-former after chemical modification
Tartaric acid	Its P-containing esters are char-formers
Tea Saponin	Carbon source Blowing agent
Nucleic acids	Intumescent systems with a predominant condensed phase action
Whey proteins	Char-formers
Caseins	Char-formers
Hydrophobins	Char-formers
Vegetable oils	Char-formers after chemical modification
Lignin	Char-former (alone or in combination with other FRs) Intumescent system after chemical modification with N-containing compounds
Phloroglucinol	Surfactant for the modification of layered double hydroxides
Cardanol	Surfactant for the modification of layered double hydroxides
Levulinic acid	Char-former after chemical modification
Phytic acid and phytates	Char-formers Components for intumescent systems

**Table 2:** Recent applications of bio-sourced flame retardants for textiles: performance indicators and main outcomes, as discussed in the present paragraph.

Textile Substrate	Type of Bio-Sourced Flame Retardant	Main Outcomes	Ref.
Cotton	Ammonium salt of arginine hexamethylenephosphonic acid	Self-extinction in vertical flame spread tests High Limiting Oxygen Index values (up to 45.1%) No ignition in forced-combustion tests (irradiative heat flux: 35 kW/m^2^) Washing fastness (50 laundry cycles)	[85]
Cotton	Ammonium starch phosphate	Self-extinction in vertical flame spread tests No ignition in forced-combustion tests (irradiative heat flux: 35 kW/m^2^) Washing fastness (50 laundry cycles)	[86]
Cotton	Graphite carbon nitride/phosphorylated chitosan (2 or 4 layer-by-layer assemblies)	Limiting Oxygen Index values up to 30.1% Decrease in the peak of Heat Release Rate (up to 56%) 90% efficiency in removing an organic dye (Rhodamine B)	[87]
Cotton	Chitosan + biochar/phytic acid (5 layer-by-layer assemblies)	High Limiting Oxygen Index values (up to 64.1%) Decrease in the peak of Heat Release Rate (by 88.3%) and Total Heat Release (by 87%) Washing fastness (10 laundry cycles)	[79]
Cotton	Laccase/Phytic acid (1 layer-by-layer assembly)	Self-extinction in vertical flame spread tests Decrease in the peak of Heat Release Rate (by 88%) and Total Heat Release (by 87%) observed in pyrolysis-combustion flow calorimetry tests	[88]
Cotton	2,6-dimethoxy polysaccharide ammonium phosphate	Self-extinction in vertical flame spread tests High Limiting Oxygen Index values (up to 49.4%) Decrease in the peak of Heat Release Rate (by 93%) and Total Heat Release (by 50%) Significant increase in smoke production Washing fastness (50 laundry cycles)	[89]
Cotton	Zeolitic imidazolate framework-8 modified with chitosan and Zn^2+^	Decrease in peak of Heat Release Rate (by 21.5%), Total Heat Release (by 16.4%), Total Smoke Release (by 16.3%), and Total Smoke Production (by 61%) Synergistic effectiveness parameter of 1.63 Antibacterial activity against *S. aureus* and *E. coli* UV-resistance	[90]
Cotton	Phosphorylated furan-based FR	Self-extinction in vertical flame spread tests (minimum FR loading: 12 wt.%) Decrease in the peak of Heat Release Rate (up to 71%) and Total Heat Release (up to 38%)	[91]
Cotton	Branched polyethylene imine/cellulose nanocrystals polyelectrolyte complex	Self-extinction in horizontal flame spread tests Durability after leaching tests performed in mild conditions	[92]
Cotton	Phytic acid + 3-(2-aminoethylamino)-propyltrimethoxysilane	Self-extinction in vertical flame spread tests Washing fastness (50 laundry cycles)	[93]
Cotton	Egg white proteins/magnesium lignosulfonate–diammonium phosphate (5 layer-by-layer assemblies)	Self-extinction in both vertical and horizontal flame spread tests Decrease in the peak of Heat Release Rate (by 60.1%), Total Heat Release (by 59.3%), and Fire Growth Capacity (by 81.5%) observed in pyrolysis-combustion flow calorimetry tests	[94]
Cotton	Lignin-silica-based liquid + 9,10-Dihydro-9-oxa-10-phosphaphenanthrene-10-oxide	Self-extinction in vertical flame spread tests (minimum FR loading: 23.2%) Decrease in the peak of Heat Release Rate (by 78%) and Total Heat Release (by 65%), observed in pyrolysis-combustion flow calorimetry tests	[95]
Cotton	Chitosan protonated with amino trimethylene phosphonic acid	Self-extinction in vertical flame spread tests (minimum FR loading: 11.5 wt.%) Limiting Oxygen Index values up to 29.7% Decrease in peak of Heat Release Rate (−87%), Total Heat Release (−62%), and Total Smoke Release (−50%), in forced-combustion tests Antibacterial activity against *E. coli* and *S. aureus*	[96]
Cotton	Sol-gel coating made of γ-ureidopropyltriethoxysilane and ammonia phytate	Self-extinction in vertical flame spread tests Limiting Oxygen Index values up to 31% Decrease in peak of Heat Release Rate (−48%), Total Heat Release (−40%), and Total Smoke Production (−48%), in forced-combustion tests	[97]
Wool	Ammonium phytate	Self-extinction in vertical flame spread tests (minimum FR loading: 7.5 wt.%) Decrease in the peak of Heat Release Rate (by about 34.1%) observed in pyrolysis-combustion flow calorimetry tests Washing fastness (15 laundry cycles)	[98]
Silk	Phytate urea salt	Self-extinction in vertical flame spread tests Decrease in the peak of Heat Release Rate (up to 41%) and Total Heat release (up to 59%) Washing fastness (35 laundry cycles)	[99]
Silk	Glycidyl phytate isocyanurate	High Limiting Oxygen Index (up to 32.5%) Decrease in the peak of Heat Release Rate (up to 46.1%), Total Heat Release (up to 29.6%), and Heat Release Capacity (up to 39.6%) in pyrolysis-combustion flow calorimetry tests Washing fastness (30 laundry cycles)	[100]
Silk	Pentaerythritol phytate ethylenediaminetetraacetic ester	High Limiting Oxygen Index (up to 35.2%) Decrease in the peak of Heat Release Rate (up to 41%) and Total Heat Release (up to 49%) observed in pyrolysis-combustion flow calorimetry tests Washing fastness (15 laundry cycles)	[101]
Silk	Coating made of phytic acid, triethanolamine, and citric acid	Self-extinction in vertical flame spread tests Limiting Oxygen Index up to 32.5% Decrease in peak of Heat Release Rate (up to 43%) and Total Heat release (up to 57%) observed in pyrolysis-combustion flow calorimetry tests Washing fastness (30 laundry cycles)	[102]
Polyester	Sodium alginate and Fe^3+^	Self-extinction in vertical flame spread tests Limiting Oxygen Index up to 29% Decrease in the peak of Heat Release Rate (up to 70%) Total Heat release (up to 12%), and Total Smoke Production (up to 27%)	[103]
Polyester	Coating made of phosphite, pentamethyldisiloxane, urea, and sodium alginate	Self-extinction in vertical flame spread tests Limiting Oxygen Index of 35.3% Decrease in the peak of Heat Release Rate (45%) Total Heat release (57%), and Total Smoke Production (48%) Acceptable washing fastness (25 laundry cycles)	[104]
Kapok	Phytic acid and urea	Self-extinction in vertical flame spread tests Decrease in the peak of Heat Release Rate (up to 69.6%), Total Heat Release (up to 78.4%) and Heat Release Capacity (up to 71.7%) in pyrolysis-combustion flow calorimetry tests Washing fastness (30 laundry cycles)	[105]
Nylon/Cotton	Chitosan/Phytic acid/Tannic acid layer-by-layer assemblies (15 quad-layers)	Self-extinction in vertical flame spread tests Decrease in the peak of Heat Release Rate (by 51%) and Total Heat Release (by 39%) observed in pyrolysis-combustion flow calorimetry tests No changes in the “hand” of the treated fabrics	[106]
Nylon/Cotton	Phytic acid and L-cysteine	Limiting Oxygen Index of 27.2% Self-extinction in vertical flame spread tests Decrease in the peak of Heat Release Rate (by 65.4%), Total Heat Release (by 62.6%), Total smoke Production (by 64.5%) and Smoke Production Rate (by 67.6%) High hydrophilicity Antibacterial activity against *E. coli* and *S. aureus*.	[107]
Polyester/Cotton	Phytic acid-urea salt	Limiting Oxygen Index of 27.3% Self-extinction in vertical flame spread tests Decrease in the peak of Heat Release Rate (by 42%) and Total Heat Release (by 39%) Poor washing fastness	[108]
Cotton/Lyocell	MXene and bio-based carbon dots	Self-extinction in vertical flame spread tests Limiting Oxygen Index up to 36% Decrease in the peak of (Heat Release Rate (by 74%) and Total Heat Release (by 28%) High electrical conductivities (up to 31.8 S m^−1^) Sensing properties Washing fastness (20 laundry cycles)	[109]

## Data Availability

No new data were created or analyzed in this study. Data sharing is not applicable to this article.
